# Severe malaria management: current situation, challenges and lessons learned from Gezira State, Sudan

**DOI:** 10.1186/s12936-019-2805-z

**Published:** 2019-05-14

**Authors:** Fahad A. Elnour, Mohammed E. A. Alagib, Devendra Bansal, Elmoubasher Abu Baker Abd Farag, Elfatih M. Malik

**Affiliations:** 1 Communicable & Non Communicable Diseases Control Directorate, Federal Ministry of Health, Nile St, Khartoum, Sudan; 2 Planning and International Health Directorate, Federal Ministry of Health, Nile St, Khartoum, Sudan; 3 Communicable Diseases Control Programmes, Public Health Department, Ministry of Public Health, Doha, Qatar

**Keywords:** Malaria, Compliance, Hospital readiness, Providers’ knowledge, Anti-malarial, Guideline, Supportive treatment, Gezira State and Sudan

## Abstract

**Background:**

The present study aimed to evaluate the management of severe malaria at Gezira State hospitals in Sudan by assessing hospital readiness, health care provider knowledge and the care received by severe malaria patients.

**Methods:**

A cross-sectional descriptive study was performed to assess the severe malaria management practices at hospitals level in Gezira State. The study population included hospitals, health care providers and patients. Data was collected using checklists and structured questionnaires.

**Results:**

A total of 20 hospitals, 158 health care providers and 370 patients were included in the study. Out of the total hospitals, 95% (19/20) were providing 24 h outpatient services, 65% (13/20) had ICU units, while triage system was found in only 35% (7/20) of hospitals. From all hospitals evaluated, 90% (18/20) were suffering from shortage of staff, especially doctors. About half of the health care providers (46.7%) did not receive severe malaria management training. The average knowledge score among health care providers was 55.4%. Microscopy was available in all hospitals (100%), while rapid diagnostic test, complete blood count and renal function test were available in 15 hospitals (75%). Fever was the most presenting symptom (97.8%) followed by repeated vomiting (51.4%), convulsion in children (24.3%) and prostration in adult (57.9%). Correctly diagnosed patients were 68.9%. Essential tests were done for only 11.1% of patients. Majority of patients (91.7%) were treated with quinine, 5.9% received artemether, while 2.4% were treated with artemether–lumefantrine. Those who received both the correct dose and dosing regimen were 53.8%. The overall compliance to guidelines was 2.2%.

**Conclusion:**

This study highlights the fact that management of severe malaria at hospital level was suboptimal with serious shortcomings in the different aspects of care particularly in specialized hospitals. Technical staff was inadequate, hospitals were anguish from defective emergency services, and most patients were not treated according to the national guidelines.

**Electronic supplementary material:**

The online version of this article (10.1186/s12936-019-2805-z) contains supplementary material, which is available to authorized users.

## Background

In spite of the remarkable progress that has been made in the fight against malaria, it remains as an important public health problem in Sudan [1]. There are more than hundred thousand severe malaria cases being reported annually. Although inpatient malaria death rate has been reduced from 8.4 deaths/100,000 population in 2000 to 1.8 deaths/100,000 population in 2016, there was no major change or slightly increased death rate during the period 2011–2016 and most of this decrease occurred between 2000 and 2010. Around one-third of malaria death is occurring among children below 5 years of age. Gezira State, the study area, is carrying significant burden of the disease where severe malaria cases represent the first cause of hospital admission and 20% of the overall severe malaria case-load in the country. In 2016, Gezira State per se accounted for about 17% of the total malaria death [2]. The transmission of malaria in this state is characterized by mainly seasonal and unstable pattern and almost all population living with low immunity and high risk of malaria, especially children below 5 years and pregnant women [3].

Three levels of health care services exist in Sudan; the primary health care level (PHC), secondary and tertiary health care levels. Malaria diagnosis and treatment usually take place at primary health care level through community health workers (CHWs), medical assistants or doctors. Although there are no clearly defined and strictly implemented referral mechanisms between the three levels, severe malaria is usually referred from the family health units and centers to be treated at hospital level. At hospital level all cases are received and admitted at outpatient clinic for 24 h and then shifted to the ward. According to the national guidelines, management of severe malaria includes; general management to the general condition of the patient and specific treatment which either by giving quinine or artemether injection. It is worth mentioning that artesunate injection has been introduced recently in the treatment policy. Pre-referral treatment using artesunate suppositories or quinine IM is also recommend for cases referred from the lower levels [4]. Moreover, artemether–lumefantrine and dihydroartemisinin-piperaquine used as first- and second-line treatment for uncomplicated malaria, respectively, and quinine or intravenous artesunate are recommended for the treatment of severe malaria in Sudan.

Prompt and effective case management is one of the key factors in malaria control. Management of malaria, as revealed in many studies, is not satisfactory largely due to poor adherence of health care providers to national policies, which is often associated with weak knowledge of care providers and poor infrastructures at health facility level [5]. This study aimed to assess the management of severe malaria at hospital level in Gezira State, through assessing hospital readiness, health care provider knowledge, and the care received by severe malaria patients.

## Methods

### Study design and population

This was a cross-sectional descriptive facility-based study conducted to assess the severe malaria management practices at hospitals level in Gezira State, Sudan using questionnaires and checklists. The study was carried out during the peak malaria transmission seasons, autumn–winter 2017. The study population includes general, specialized and rural hospitals providing severe malaria management; health care providers directly responsible for making decisions of patients care; and patients admitted to hospitals and diagnosed as severe malaria.

### Sample size and sampling

Out of the total hospitals in Gezira State, twenty hospitals fulfilled the inclusion and exclusion criteria were included in the study. For health care providers, 158 were selected using the equation for calculating sample size for a finite population. Regarding severe malaria patients a sample size of 370 patients was estimated, assuming 70% of patients with severe malaria are appropriately treated, at 95% confidence level, with a tolerable error of 0.05% and 15% non-response rate. Hospitals were selected from the list of hospital in the state. All hospitals meeting the inclusion and exclusion criteria were included in the study. As a result 20 hospitals were selected; among them eight were general, seven specialized (four paediatrics and three obstetrics) and five rural hospitals. These hospitals were distributed across all localities in Gezira State. The health care providers were selected using non-probability sampling from those available at these hospitals during the study period till satisfaction of the sample size. Health care providers selected were those responsible for making the decision on patient management, namely doctors at different professional levels. Similarly, for collecting data from severe malaria patients; all those admitted with sever malaria during the period of study were enrolled, till reached the total number required. Patients were interviewed when they are in a good condition and eligible to respond or otherwise the caregivers respond on behalf of them, as well as reviewing patients’ records.

### Data collection and study variables

Considering the fact that Gezira State has eight localities, a supervisor from each locality was nominated, informed about the purpose of the study and trained by the researcher on how to conduct the data collection. The supervisors selected two data collectors from each hospital and trained them on how to use the questionnaire and check lists and how to approach target groups. The process started in each hospital by meeting with hospital manager, explaining the purpose of the study and taking permission to start the data collection in the hospital. The readiness of these hospitals, the knowledge of health care providers working for them and care received by the patients were assessed using the specific tools designed for the purpose of this study.

Data was collected using three types of data collection tools adapted from the World Health Organization (WHO) hospital assessment tool, results and lessons learnt from implementation of WHO assessment tool and the checklist for assessing management of severe malaria [6–8]. The first tool was hospital check list which was designed to be filled by hospital managers aiming to assess the hospitals’ readiness in the various dimensions; including staffing, infrastructure, outpatient and inpatient facilities, laboratory services and supply system in the hospital (see Additional file 1). The second tool was self-administered questionnaire to assess knowledge of health care providers, which included three scenarios; paediatrics, adult and pregnant women cases. The scenarios were covering clinical assessment, investigations, and management and follow-up aspects. The third tool was the patient’s questionnaire, which includes two parts; the first was information taken from patient; clinical history, management received, and satisfaction of the service provide, and the other part was medical record review; in terms of completeness and content in light of the guidelines for severe malaria management from history taking, through investigation and treatment to follow-up.

### Data management and analysis

After collection, data was reviewed for completeness, verified and cleared by the researcher. The data was entered in Microsoft excel and analysis was carried out using SPSS (version 23). Descriptive data was summarized in percentages, frequencies and ratios and presented in tables and charts.

### Study definitions

The assessment in this study was conducted based on the following explanations; (i) *Availability of essential equipment*: availability of one piece or more from each type of equipment; (ii) *Presence of triage system*: sorting out of patients attending at hospital outpatient clinics by dedicated technical staff; (iii) *Correctly diagnosed patient*: when there was documented fever (or history of fever) with a positive malaria test and at least one feature of severe disease; (iv) *Correct anti*-*malarial medicine prescribed*: administration of parenteral quinine, artemether or artesunate; (v) *Correct anti*-*malarial drug dose and dosing regimen*: intravenous quinine 10 mg/kg every 8 h or intramuscular artemether 3.2 mg/kg on day 1, followed by 1.6 mg/kg; (vi) *Appropriately treated patients*: if patients received the correct anti-malarial medicine, at the right dose and dosing regimen; (vii) *Compliance to guidelines*; detection/eliciting severe malaria features (at least one); (viii) *Conducting essential tests*: malaria test, blood glucose and haemoglobin), prescription of correct AMDs, prescription of correct dose and dosing schedule (regimen), giving supportive treatment (I.V fluids) and follow-up done; and (ix) good referral practice: the patient referred with referral note and received pre-referral anti-malarial treatment. To evaluate the knowledge of health care providers through the three clinical scenarios, the responses of participants were evaluated. Equal score was given for each case scenario and for the three sections of each case scenario. The total score of each case scenario is 12°, with 4° for every section in the scenario (symptoms and signs, investigation and management including follow-up). Based on the overall score and since sufficient knowledge is mandatory for dealing serious problems like severe malaria, the knowledge of health care providers was classified as acceptable if the score was 90% or above, otherwise it was considered as unacceptable. Severe malaria is defined as a medical emergency which requires hospitalization, and the clinical manifestations include impaired consciousness (cerebral malaria), prostration, multiple convulsions, deep breathing and respiratory distress, acute pulmonary oedema. The laboratory findings include severe anaemia (Hb < 5 g/dl), hypoglycaemia (< 40 mg/dl), metabolic acidosis, hyperparasitaemia (250,000/μl), hyperlactaemia (lactic acidosis); and renal impairment.

### Ethical considerations

Ethical approval was obtained from the Sudan Medical Specialization Board (SMSB). Permission was taken from Gezira State Ministry of Health, Sudan and from the manager of each hospital. Due to the fact there is a cultural barrier to obtain written consent; verbal informed consent was obtained from health care providers and patients or caregivers prior to completing the questionnaire and interview at the selected hospitals. All measures to maintain confidentiality and anonymity of data were put in place.

## Results

In this study 20 hospitals in Gezira State were assessed for their readiness and 158 health care providers were interviewed. In addition, the care received by 370 patients admitted at these hospitals during the period of the study was reviewed.

### Hospitals profile, management and readiness

Out of twenty hospitals; five general, seven specialized and eight were rural hospitals. The result revealed that most of hospitals (95%, n = 19 and 85%, n = 17) were having 24 h outpatient services and 85% (17/20) of hospitals containing an emergency room for admission of patients in the first 24 h. The intensive care unit (ICU) for the management of critically ill patients was found in 65% (n = 13) of hospitals. Triage system for sorting-out of critically ill patients was found in only 35% (7/20) of hospitals.

Hospitals’ readiness was assessed in terms of availability of staff, essential equipment, laboratory services and medical supplies. In regard to hospital staff adequacy, doctors were reported to be inadequate in the majority (90%, 18/20) of hospitals. Nurses and laboratory technicians were found to be adequate only in 25% (5/20) of hospitals. Sphygmomanometer was found in all hospitals, while thermometer in only 40% (8/20) of hospitals (Table 1). Microscopy as a gold standard malaria test was found to be available in all selected hospitals. Rapid diagnostic test (RDT) as alternative for microscopy was available in 75% (15/20) of hospitals (Table 1). During the time of the study quinine injection was available in majority (95%, n = 19/20) of the hospitals. Artemether–lumefantrine and quinine tabs were found to be available in 15% (3/20) and 85% (17/20) of hospitals, respectively (Table 1).

**Table 1:** Hospital readiness variables in Gezira State, Sudan

Variables	Hospitals (N = 20) n (%)
Availability of equipment	
Sphygmomanometer	18 (90%)
Stethoscope	17 (85%)
Oxygen in cylinders	18 (90%)
Weighing scale	12 (60%)
Thermometer	11 (55%)
Availability of tests	
Functioning microscopy	20 (100%)
Blood glucose test	20 (100%)
Malaria rapid diagnostic tests	15 (75%)
CBC test	15 (75%)
RFT	15 (75%)
X-ray	9 (45%)
ABG (arterial blood gases)	3 (15%)
Blood lactate level	0 (0.0%)
Anti-malarial medicine available on the day of survey	
Quinine inj	18 (90%)
Artemether inj	14 (70%)
Quinine tabs	17 (85%)
Coartem tabs	3 (15%)

### Knowledge and training of health care providers

A total of 158 doctors of different levels (house officers, medical doctors, registrars and consultants) participated in the study. About half of them (46.7%) reported that they did not receive any training on severe malaria management. In response to the three hypothetical case scenarios; paediatric, adult and pregnant women, the average score for all health care providers was found to be 55.4%. The average score of each type of health care providers was in all scenarios was 51.8%, 57.8%, 60.4%, and 44.5% for house officers, medical officers, registrars and consultant, respectively. The performance of health care providers varied across the different sections of the three case scenarios with a mean score of 59%, 57.9%, 49% and 33% for knowledge about; features of severe malaria, investigations, treatment and follow-up, respectively. Those who scored within the acceptable level of knowledge (≥ 90%) about features of severe malaria were 19% for, investigations were 15%, and treatment and follow-up were 2%. The overall score of health care providers within the acceptable level of knowledge in all case scenarios was only about 2%.

### Clinical practice related to severe malaria management

A total of 370 participants were included and two-third (66.4%) of them were children; about one-third of them were under 5 years, while one-third (33.6) were adults. Around 56% were females, and about 59% of participants were urban population. The median of duration of stay at hospital was 2 days.

Out of the total participants, 56.2% were referred to hospital from other health facilities, 39.4% were given a referral note and 20.9% received pre-referral anti-malarial treatment. Good referral practice was found in only 11.1% of referred patients. From those who reported that they were brought in a critical condition (17.1%), 82.9% were given a priority in receiving care. Only 30% of patients reported having received the medical care within 30 min of their attendance.

Most of patients were asked about their age (70%), fever or history of fever (93.8%) and continuous vomiting (58.6%). However, many common serious manifestations of severe malaria were not regularly checked. Repeated convulsions; prostration and difficult breathing were elicited in only in 39.2, 36.5% and 28.6%, respectively. Weight was measured in around two-third of children, temperature in 63% and blood pressure in 34.1% (Table 2).

**Table 2:** Frequency of features of severe malaria management in the hospitals of Gezira State, Sudan

Features of severe malaria	Frequency (N = 370)	%
Symptoms		
Fever (or history of fever)	347	93.8
Repeated vomiting	217	58.6
Convulsions	145	39.2
Refusal of feeding (in children)	88	36.8
Generalized body weakness	135	36.5
Yellow eyes (jaundice)	132	35.7
Cough	128	34.6
Loss of consciousness	112	30.3
Rapid/difficult breathing	106	28.6
Signs		
Pulse rate	291	78.6
Respiratory rate	276	74.6
Weight (in children)	169	70.7
Comment on signs of anaemia	236	63.8
Temperature	233	63.0
Jaundice	210	56.8
Level of consciousness	168	45.4
Signs of dehydration	141	38.1
Blood pressure	126	34.1
Neck stiffness	101	27.3
Weakness	96	25.9

Microscopy was requested for the majority of the patients (94.9%) to confirm malaria, 98.5% revealed positive result. Blood glucose level was requested for 27.8% of patients out of them 12.7% were hypoglycaemic. The haemoglobin level was ordered for 49.5%, almost half of them (46.8%) were anemic and 5.6% were severely anemic. Correctly diagnosed patients were 68.9%. The majority of patients were treated mostly with quinine (91.7%), 5.9% received artemether, while 2.4% were treated with artemether–lumefantrine. Around two-third of patients (71.6%) received the right dose of anti-malarial drug, whether quinine or artemether, in 57.3% the dosing regimen is also correct. About one-fifth of patients (18.4%), all of them received quinine; the drug was prescribed twice a day instead of three times a day. Those who received both the correct dose and dosing regimen were 53.8%. The follow-up at least for temperature, blood pressure and respiratory rate was done for 60%. Overall, those who were appropriately treated, according to guidelines, were found to be only 2.2%. The majority of patients (90.8%) paid for at least some of the medicine prescribed. Around two-third of them (66.8%) obtained the supplies through out of pocket payment, while those who paid for anti-malarial drugs were 15.9%.

## Discussion

The findings of this study revealed that management of severe malaria at hospital level was substandard. Major defects were detected in the general setup and readiness of hospitals, availability of supplies and the level of care provided. This study revealed that the triage system, as one of the parameters that indicate quality and inclusiveness of services provided, was lacking in most of the hospitals. This is particularly important since many patients seek medical advice usually after development of complications and deterioration of their condition. In a study conducted Sudan, 73% of those who sought medical advice did so after the first day of start of the illness [9]. In addition, most of patients in this study were referred to hospital without receiving pre-referral anti-malarial treatment and many of them spent long time to receive care. These facts could provide some explanation for the high number deaths occurring each year in Sudan.

The current status of hospitals demonstrated that almost all hospitals are suffering from clear shortage of staff, (especially doctors) and equipment. Since severe malaria is a medical emergency with multisystem disorders, full set of laboratory services is required in every hospital that deals with severe malaria patients.

Although malaria microscopy and blood glucose tests were consistently available in all hospitals, other important test such renal function and complete blood count were lacking in a quarter of hospitals. These findings were also reported from studies conducted in similar settings [10, 11]. ABG was found only in one-sixth of hospitals, while none of them conduct serum lactate level test in spite of that fact that it has been found as an important marker for the prognosis of patients admitted with severe malaria [12]. Although this study was not designed to assess the effect of hospital readiness on clinical outcome, these shortcomings are expected to affect quality of care and consequently patient survival [13].

Considering availability of severe malaria drugs, though the majority of hospitals showed availability of quinine in its different forms at the time of study; all anti-malarial drugs, including oral continuation therapies, experienced stock out during the last 3 months before study. The problem of availability of drugs and supplies could be attributed to the weak, fragmented supply system that delivers the commodities from the central level down to the facility level and also lack of accountability. This result may explain the fact that many patients paid for anti-malarial drugs and other supplies.

In this study, knowledge of doctors about severe malaria management was far below expectations. The average knowledge across the three different scenarios; paediatric, adult and pregnant women was below the acceptable level. Unexpectedly, the consultants, together with house officers, have shown the lowest scores. While the level of knowledge about manifestation of severe malaria and investigation was relatively adequate, the awareness about management and follow-up appeared to be the weakest part. These observations corroborate previous report from Angola, where the average knowledge of health care providers was found to be only 5.6% [11]. All these facts highlight the immense gap in the knowledge about severe malaria among health care providers, which is expected to seriously affect handling severe malaria patients safely and effectively. This study also revealed that half of health care providers did not receive any training on severe malaria management, which could be one of the explanations of low knowledge among health care providers. The low performance of house officer and consultants could be due to the production of enormous number of house officers but very limited training opportunities, while for consultants besides the fact that many of them were reluctant to follow protocols, in most of the cases they are not taken into account when planning training programmes. To upgrade the performance of health care providers; expansion of training programmes and improving their quality through reviewing the materials and methodologies of implementation is extremely required.

When patient’s care was evaluated, systematic checking of severe malaria manifestations was found to be far below the required level. Apart from fever, the other symptoms were not checked for in many patients. Rapid/difficult breathing and generalized body weakness (prostration) although they are associated with poor prognosis and need special attention [4, 14], was explored in only 28.6% and 36.5% of patients. In one-third of children records contained no weight measurements, documentation of temperature and signs of anaemia appeared in less than two-third of patients records and more seriously blood pressure measurement is missing in almost two-third of patients. These facts could be due to either poor documentation or defective patient care with missing of life threatening complication, which is in turn jeopardize the subsequent steps of patient management.

In this study, microscopy, a gold standard test, was used to confirm malaria in most of the cases. However, in previous studies from Swaziland and Zimbabwe, RDT was used to confirm malaria in the majority of cases [14, 15]. Almost all patients in this study were tested positive and only 2 cases tested negative. Those who tested negative were treated as severe malaria cases; this was most likely based on clinical judgment, though this is sometimes needed but it may lead to missing of other serious illnesses (e.g. meningitis) and inappropriate management of the patient. It worth mentioning that; blood glucose test was ordered for only 27.8%. Similar study conducted in Kenya revealed that investigations other than malaria test like blood glucose level was done in only 2.6% of cases [16]. Since hypoglycaemia is very likely with severe malaria and can be caused by many factors, blood glucose test has to be made essential for any patient with severe malaria [17, 18]. In spite of the fact that severe anaemia is common presentation of severe malaria and is a leading cause of death, especially in children haemoglobin level was requested for only less than half of patients. Anaemia was found in more than two-third of recorded results with more than 10% of anemic patients had severe anaemia. It is obvious from the current results that management practice of severe malaria was focusing only on giving the anti-malarial drugs with little attention to the management of the complications that could arise. This malpractice could be improved by increasing the frequency of training programmes to cover all targeted care providers. In addition to that, implementation of supportive supervision represents a very important element in improving the clinical practice and adherence to malaria guidelines.

The majority of patients in the present study were treated with the recommended anti-malarial drug for severe malaria and similar findings were observed in the previous studies from some countries in the region [14, 16, 19]. However, 8 patients (2.4%) in this study were treated with artemether–lumefantrine; this was considered a major violation of the treatment guidelines since it put the life of these patients at risk of death. Although the majority of patients were treated with recommended anti-malarial drugs, only 53.8% received the correct dose and right dosing regimen. Some patients received quinine injection twice a day instead of three times a day, which is not complying with the guidelines. Generally, the use of the correct anti-malarial drugs for management of severe malaria appeared to be good; this could be due to presence of clear guidelines, training programmes for providers and free distribution of severe malaria treatment. Furthermore, the study revealed that significant proportion of patients paid for anti-malarial drugs or related medical supplies. This fact may hinder some patient to receive the full course of management at hospital, and in many cases, it will represent a real barrier to seek medical advice and look for alternative solutions as revealed in the study conducted in Sudan [20].

## Conclusion

The present study highlights the fact that management of severe malaria at hospital level was suboptimal with serious shortcomings in the different aspects of care. Those who were appropriately treated for severe malaria with all six parameters properly done from patient assessment to follow-up, the overall compliance with guidelines, were found to be only 2.2%. Similarly, in Uganda, only 16.9% of patients were considered treated appropriately for severe malaria [10].

### Recommendations

1. Ministry of Health (MoH) needs to review hospitals’ readiness and setup and ensure availability of equipment, essential laboratory tests and adequate, continuous supply system.
2. MoH should develop better strategies to improve health worker adherence to guidelines; through expansion of training programmes on severe malaria and referral guidelines including pre-referral treatment for hospital staffs (clinicians, nurses and laboratory technicians) and lower level staffs.
3. It is also important to ensure the quality of trainings being provided by reviewing and updating the training materials and methods.
4. MoH and hospital directorates has to establish malaria mortality audit at hospital level with the aim of improving clinical practice related to malaria and reduce malaria deaths.
5. It is necessary to accelerate the introduction of artesunate injection as a treatment of choice for severe malaria management and review the implementation of free malaria treatment policy.

## Additional file

**Additional file 1.** Checklist for each health facility, questionnaire for health care providers and patients/caregivers.

## Data Availability

Yes—all data are fully available without restriction and from the corresponding author on reasonable request.

## Abbreviations

RDT: rapid diagnostic test
WHO: World Health Organization
SMSB: Sudan Medical Specialization Board
ICU: intensive care unit
ABG: arterial blood gases
