# Supplementary material for: Severe malaria management: current situation, challenges and lessons learned from Gezira State, Sudan
Source: Malar J. 2019 May 14;18:170. doi: 10.1186/s12936-019-2805-z (PMC6515598; doi:10.1186/s12936-019-2805-z)
Supplement: Supplementary file 1 — Additional file 1. Checklist for each health facility, questionnaire for health care providers and patients/caregivers. [file 12936_2019_2805_MOESM1_ESM.docx]

**ANNEXES**

**Annex (1): Checklist for each Health Facility**

***Note:***

*Complete section 1, 2 and 3 by interviewing the medical director; and the most relevant staff head for other sections.*

1. **General information:**
2. Locality name: ………………………………………………………………….
3. Name of hospital: …………………………………………………………..
4. Type of hospital
5. General hospital
6. Rural
7. Specialized
8. **Staffing:** Complete the following information

| **Type of staff** | **Number of staff required according to MOH standards** | **Number of staff employed** | **Number of staff scheduled for duty today** | **Number of staff available on duty today** | **The number of employee received training on severe malaria management** |
| --- | --- | --- | --- | --- | --- |
| Specialist |  |  |  |  |  |
| Registrar |  |  |  |  |  |
| Medical Doctor |  |  |  |  |  |
| Nursing staff (nurses, … ) |  |  |  |  |  |
| Laboratory technicians |  |  |  |  |  |
| Laboratory assistants |  |  |  |  |  |
| Pharmacist/assistant pharmacist |  |  |  |  |  |
| Other (Specify) |  |  |  |  |  |

**Contact No. of medical director:** ………………………………………………………

1. **Patient care system setup**
2. Is there a 24 hrs outpatient services available? Yes ( ) No ( )
3. Is there an emergency ward for admission of patients in the first 24hrs?

Yes ( ) No ( )

1. Is there an ICU for admission of critically ill patients? Yes ( ) No ( )
2. Is there an outpatient sorting out approach to select severely ill patients for urgent care? Yes ( ) No ( )
3. Do Lab request for critically ill patients given priority? Yes ( ) No ( )
4. **Outpatient/inpatient facilities:** If available/functional put (√ ), if not put (**×)**

|  | **Item** | **Available at outpatient put** | **Available at ward** |
| --- | --- | --- | --- |
| **Equipment:** at least one item available | | | |
|  | Thermometer |  |  |
|  | Weighing scale |  |  |
|  | Stethoscope |  |  |
|  | Sphygmomanometer |  |  |
|  | Oral airways |  |  |
|  | Ambubag |  |  |
|  | NG tube |  |  |
|  | Blood transfusion sets |  |  |
|  | IV cannula |  |  |
|  | Syringes |  |  |
|  | Oxygen in cylinders |  |  |
| **Records and job aids** | | | |
|  | File for keeping record |  |  |
|  | Nursing registration book |  |  |
|  | Job aids (protocol booklet or wall charts or table charts …) |  |  |

1. **Laboratory facilities:** which of the following diagnostic facilities are available and functional **within** the hospital selected? If available put (√ ), if not put (**×)**

| **Test** | **Available** | **Functional** |
| --- | --- | --- |
| 1. Malaria rapid diagnostic tests |  |  |
| 1. Microscopy |  |  |
| 1. Blood glucose test |  |  |
| 1. Urine analysis |  |  |
| 1. CBC test |  |  |
| 1. RFT |  |  |
| 1. X-ray |  |  |
| 1. ABG (arterial blood gas) |  |  |
| 1. Blood lactate level |  |  |

1. **Pharmacy:** Are the following supplies available at hospital? If available put (√ ), if not put (**×)**

| **Drug** | | **Available now** | **Available throughout the last 3 months** |
| --- | --- | --- | --- |
|  | Quinine injection |  |  |
|  | Quinine tabs |  |  |
|  | Artemether or Artesunate injection |  |  |
|  | Artemether-lumefantrine tabs |  |  |
|  | AS/SP tabs |  |  |
|  | Artemether injection |  |  |
|  | Diazepam injection |  |  |
|  | Paracetamol tabs |  |  |
|  | Paracetamol syrup |  |  |
|  | Paracetamol suppositories |  |  |
|  | Phenobarbitone tabs |  |  |
|  | Furosemide injection |  |  |
|  | Ceftriaxone or equivalent |  |  |
|  | Dextrose 5% |  |  |
|  | Dextrose 10% |  |  |
|  | Dextrose 50% |  |  |
|  | Saline 0.9% |  |  |
|  | Ringer lactate |  |  |
|  | Water for injection |  |  |
|  | Syringes |  |  |

***Date***: ….. / ……… / 2017

***Time*** ……… am /pm

**Completed by (name):** ………………………………….

**Annex (2): Health care provider questionnaire**

1. **General information:**
2. Locality name: ……………………………..………………………………………………………………
3. Name of hospital: …………………………………………………………………………………………
4. Type of hospital
5. General hospital
6. Rural
7. Specialized
8. health care provider job:
9. House officer
10. Medical officer
11. Registrar
12. Consultant
13. For how long you have been in this job? ………………………………………………………….
14. Did you receive training on guidelines of malaria management?

Yes ( ) No ( )

**Scenario (1)**

| Mariam, a 3 year old girl from a remote community in Gezira state, develops fever and convulsion (3 attacks in past 6 hrs). Later, on the evening of the same day, Mariam's speech became less comprehensible and shortly afterwards was no longer responding to any call. The mother took 2 hours to get to the nearest hospital and the child remained unconscious when they got there. In addition to loss of consciousness she was found on examination to be severely pale, jaundiced with a temperature of 38.50 C, Hb was found to be 4.5mg/dl and blood glucose was 60 mg/dl. |
| --- |

1. Could it be severe malaria?

**Answer;** yes No Don’t know

1. If the answer is yes, please answer the following questions:
2. Mention the manifestations of severe malaria in this case?
   - 1. ……………………………………………………………………………..………………………………………..
     2. ………………………………………………………….…………………………………………………………..
     3. ………………………………………………………….…………………………………………………………..
     4. ………………………………………………………….…………………………………………………………..
     5. ………………………………………………………….…………………………………………………………..
     6. ………………………………………………………….…………………………………………………………..
     7. ………………………………………………………….…………………………………………………………..
3. Mention four important laboratory tests you will do?
   1. ……………………………………………………..……………………………………………………………..
   2. ……………………………………………………..……………………………………………………………..
   3. ……………………………………………………..……………………………………………………………..
   4. ……………………………………………………..……………………………………………………………..
4. What specific treatment would you give?
   1. Option1: …………………………………………………………………………………………….……

…………………………………………………..…………….………………………………………………

1. Option2: ………………………….……………………………………………………………………….
2. If the weight of this patient is 15 kg, please determine exactly the dose and dosing schedule?

……………………………………………………………………………………………………………………

……………………………………………………………………………………………………………………

……………………………………………………………………………………………………………………

1. Mention four important parameters (clinical and laboratory) you will follow during patient admission to see the effect of management?

……………………………………………………………..…………………………………………………..

……………………………………………………………..…………………………………………………..

……………………………………………………………..…………………………………………………..

……………………………………………………………..…………………………………………………..

……………………………………………………………..…………………………………………………..

**Scenario (2)**

Nadir, a 25 years man, brought to the outpatient department of Wad Madani hospital. He became ill three days ago, with chills, sweating and headaches. He took antibiotics and anti-malaria in form of tabs with no improvement. This morning he became very ill and confused. His family brought him to your hospital.

On examination he is unconscious, axillary temperature is 40.2^0^ C; pulse 120 beats /min, regular; blood pressure 90/70 mmHg. No neck stiffness, pallor or rash. Blood film revealed malaria parasites (++++).

1. Could it be severe malaria?

**Answer;** yes No Don’t know

1. If you diagnosed this case as severe malaria, please answer the following?
2. What findings mentioned in the case were in favor of that?
3. ………………………………………………………………………………..…………………………………………
4. ………………………………………………………………………………..…………………………………………
5. ………………………………………………………………………………..…………………………………………
6. ………………………………………………………………………………..…………………………………………
7. Could you please mention other five features of severe malaria?
8. ………………………………………………………………………………..…………………………………………
9. ………………………………………………………………………………..…………………………………………
10. ………………………………………………………………………………..…………………………………………
11. ………………………………………………………………………………..…………………………………………
12. ………………………………………………………………………………..…………………………………………
13. ………………………………………………………………………………..…………………………………………
14. ………………………………………………………………………………..…………………………………………
15. Mention four important laboratory tests you will do.
16. ……………………………………………………………………….…………………………………………………
17. ……………………………………………………………………….…………………………………………………
18. ……………………………………………………………………….…………………………………………………
19. ……………………………………………………………………….…………………………………………………
20. ……………………………………………………………………….…………………………………………………
21. What are the immediate actions you will do for this patient?
22. ……………………………………………………………………………….…………………………………………
23. ……………………………………………………………………………….…………………………………………
24. ……………………………………………………………………………….…………………………………………
25. ……………………………………………………………………………….…………………………………………
26. ……………………………………………………………………………….…………………………………………
27. ……………………………………………………………………………….…………………………………………
28. ……………………………………………………………………………….…………………………………………
29. ……………………………………………………………………………….…………………………………………

**Scenario (3):**

| Salwa, a 35 year old woman, is brought to the outpatient department of the central hospital in the capital. She is a local resident, and is pregnant (20 week’s gestational age). The patient became ill three days ago, with fever, chills, sweating and headaches. Today she has been referred to your hospital because of deep labored breathing and confusion. Examination reveals a semiconscious woman, who is unable to talk. There is no neck stiffness, jaundice or pallor. Axillary temperature is 39^O^c, pulse 90 beats/ min, blood pressure 110/70 mmHg. Skin examination showed petechial hemorrhage. Plasma bicarbonate level was 24mmol/L and venous lactate level was 8mmol/L. |
| --- |

1. Could it be severe malaria? Yes No Don’t know
2. If the answer is yes, please answer the following?
3. What findings mentioned in the case were in favour of that?
4. ……………………………………………………………………………………………….………………………………..
5. ……………………………………………………………………………………………….………………………………..
6. ……………………………………………………………………………………………….………………………………..
7. ……………………………………………………………………………………………….………………………………..
8. Could you please mention four important complications that could happen?
9. …………………………………………………………………………………….…………………………………………
10. …………………………………………………………………………………….…………………………………………
11. …………………………………………………………………………………….…………………………………………
12. …………………………………………………………………………………….…………………………………………
13. Mention four important laboratory tests you will do?
14. …………………………………………………………………………………..…………………………………………
15. …………………………………………………………………………………..…………………………………………
16. …………………………………………………………………………………..…………………………………………
17. …………………………………………………………………………………..…………………………………………
18. If the whole blood glucose is 1.2 mmol/l (21.6 mg/dl), what treatment will you give?

……………………………………………..……………………………………………………..……………………………………………………….

……………………………………………..……………………………………………………..……………………………………………………….

……………………………………………..……………………………………………………..……………………………………………………….

……………………………………………..……………………………………………………..……………………………………………………….

1. If the blood film shows *P. falciparum* rings “++++” (*hyperparasitaemia*), what antimalarial drug will you administer and by what route?

…………………………………………………………………………………………………….……………………………………………………

…………………………………………………………………………………………………….……………………………………………………

…………………………………………………………………………………………………….……………………………………………………

1. What is your alternative?

…………………………………………………………………………………………………….……………………………………………………

…………………………………………………………………………………………………….……………………………………………………

…………………………………………………………………………………………………….……………………………………………………

**Annex (3): Patient/caregiver questionnaire**

*Date* ………………………………….

**A. General information:**

1. Locality name: .....................................................................................................

2. Name of hospital: ............................................................................................

3. Code of hospital: .............................................................................................

4. Patient’s name: ................................................................................................

5. Patient’s age: ........................................................................

6. Residence: ................................................................................................. 7. Sex Male ( ) Female ( )

8. Occupation: ………………………………………………….…………….

9. Patient’s level of education:

1. Informal ( )
2. Primary school ( )
3. Higher secondary school ( )
4. University and above ( )

10. Information provided by: Patient ( ) Caregiver ( )

11. Relation to the patient in case of caregiver.........................................................................

12. How long is your stay in hospital ………………..days or ………………….hrs.

**B. Patient medical history** (ask the patient or caregiver about the following questions)

1. Considering the list below, ask the patient or caregiver about what complaints brought him/her to the hospital? Check if he/she had other symptoms of severe malaria?

| **Symptoms** | **Put (**√**) if the symptom is one of the complaints** |
| --- | --- |
| Fever (or history of fever) |  |
| Continuous vomiting |  |
| Repeated convulsions |  |
| Loss of consciousness |  |
| Generalized body weakness |  |
| Rapid / difficult breathing |  |
| Refusal of feeding (in children) |  |
| Yellow eyes (jaundice) |  |
| Cough |  |
| Ear discharge |  |
| Neck stiffness, |  |
| Others (specify)…………………………… |  |
| ………………………………………… |  |

1. Do you/your patient have any underline illness? Yes ( ) No ( )
2. If yes, what is the illness …………………………………………………..
3. Did you/your patient receive any medications before coming to hospital?

Yes ( ) No ( )

1. If yes, what is the drug …………………………………………………..
2. Were you/your patient brought to the hospital in a critical condition? Yes ( ) No ( )
3. Did you/your patient given a priority in receiving the consultation? Yes ( ) No ( )
4. How long did you/your patient wait before receiving medical advice? Immediately ( ) after short time ( ) after long time ( )
5. Estimate time (if possible)..................... minutes.
6. Were you referred to the hospital from any other health facility? Yes ( ) No ( )
7. If the answer is yes, have you given a referral note? Yes ( ) No ( )
8. Did receive a pre-referral antimalarial treatment? Yes ( ) No ( )
9. When was the last attack of malaria (the one before this time) ………………………….

**Management:**

1. Did you/your patient given any injectable antimalarial at hospital here?

Yes ( ) No ( ) Don’t know ( )

1. If yes:
   1. What was the name of the injection?

………………………………………………….……………………………………………....

- 1. What was it for?

………………………………………………………………………….……………………….

- 1. How was it given?
     1. Intravenous (In a drip) ( )
     2. On the buttocks ( )
     3. On the thighs ( )
     4. Not sure ( )
  2. How many of these injections were you given per day?

……………………..……………………………………………………………………………..

- 1. For how many days were it given/ prescribed?

…………………………………………………………………………………………………..

1. Did you buy any of the following medicines because it was not available in hospital? And how much did you spent? If yes put (√ ), if no put (**×)**

| **The drug** | (√ ) or (**×)** | **The cost** |
| --- | --- | --- |
| Injectable antimalarial |  |  |
| Oral antimalarial |  |  |
| Antibiotics |  |  |
| Antipyretics |  |  |
| Supplies (I.V fluids, syringes, plaster … |  |  |
| Others ………………. |  |  |

1. Were you given enough time to explain your complaints?

Yes ( ) to some extent ( ) No ( )

1. Were you given a time to ask questions?

Yes ( ) to some extent ( ) No ( )

1. Did the provider explain to you the condition and plan of management?

Yes ( ) to some extent ( ) No ( )

1. Did the staff dealt with you in a good manner?

Yes ( ) to some extent ( ) No ( )

1. Generally, are you satisfied about the services you received?

Yes ( ) to some extent ( ) No ( )

| **Record review:** | | | |
| --- | --- | --- | --- |
| Please indicate by putting (√) sign if item was commented on in the medical records. | | | |
| **1. History: Put (**√**) if the item was recorded** | | | |
| **1.1. information/symptom** | | | |
| a)      Age | | |  |
| b)      Fever (or history of fever | | |  |
| c)      Repeated vomiting | | |  |
| d)      Convulsions | | |  |
| e)      Loss of consciousness | | |  |
| f)       Generalized body weakness | | |  |
| g)      Rapid / difficult breathing | | |  |
| h)      Refusal of feeding (in children) | | |  |
| i)        Yellow eyes (jaundice) | | |  |
| j)        Cough | | |  |
| k)      Others (specify)…………………………… | | |  |
| **1.2 Patient medical history** | | | |
| a)      Past medical history | | |  |
| b)      Feeding history | | |  |
| c)      Gestational history (in case of pregnant ladies) | | |  |
| d)      History of medicines taken | | |  |
| e)      Write down medicines recorded by the provider  ……………………………………………………………………………………………………………………..………………… | | |  |
| f)       Hypersensitivity to any drug | | |  |
| **2. Examination: Put (**√**) if the item have been commented on** | | |  |
| 1. Weight (in children) (write weight in Kg = ……………….) | | |  |
| 2. Temperature | | |  |
| 3. Pulse rate | | |  |
| 4. Respiratory rate | | |  |
| 5. Blood pressure | | |  |
| 6. Level of consciousness | | |  |
| 7. Signs of dehydration | | |  |
| 8. Comment on signs of anemia | | |  |
| 9. Jaundice | | |  |
| 10. Neck stiffness | | |  |
| 11. weakness | | |  |
| 12. Comment on neck stiffness | | |  |
| 13. Chest exam | | |  |
| 14. Abdomen | | |  |
| 15. Skin | | |  |
| **3. Investigations: What investigations have been requested? Are all requested investigations documented?** | | | |
|  |  |  |  |
| **Investigation** | **Ordered** | **Recorded** | **Result** |
| Blood film for malaria |  |  |  |
| Blood glucose |  |  |  |
| Hemoglobin |  |  |  |
| CBC (complete blood count) |  |  |  |
| Renal function |  |  |  |
| Urine analysis |  |  |  |
| Chest x-ray |  |  |  |
| CSF (water from the back) |  |  |  |
| **4. Management: Were diagnoses recorded? What medicines have prescribed and how?** | | | |
|  | | | **Recorded** |
| Provisional diagnosis | | |  |
| Final diagnosis | | |  |
| IV fluids | | |  |
| Dextrose | | |  |
| Blood transfusion | | |  |
| Nasogastric tube | | |  |
| Quinine IV or IM | | |  |
| Quinine tablets | | |  |
| Ceftriaxone | | |  |
| Coartem | | |  |
| Others (specify)…………………………………… | | |  |
| Write dosing and schedule if antimalarial ordered? | | | |
| **5. Follow up of patient:** | | |  |
| Follow is part of plan management | | |  |
| There is regular follow up | | |  |
| There is at least daily follow up | | |  |
| **The following are being followed:** | | |  |
| Vital signs (BP, Pulse, RR) | | |  |
| Level of consciousness | | |  |
| Blood glucose | | |  |
| Level of parasitaemia | | |  |
